# Four-year outcomes of full-arch fixed dental prostheses using CAD/CAM frameworks: A retrospective review of 15 cases

**DOI:** 10.4317/jced.55176

**Published:** 2018-10-01

**Authors:** Ilser Turkyilmaz, Niki-Haj Hariri

**Affiliations:** 1 DMD, PhD, Clinical Associate Professor, New York University College of Dentistry, Department of Prosthodontics, New York, NY, USA; 2 Fourth-year Dental Student, New York University College of Dentistry, New York, NY, USA

## Abstract

The aim of this report is to analyze the clinical performance of 20 full-arch implant-supported titanium frameworks using CAD/CAM (Computer-Aided Design and Computer-Aided Manufacturing) technology. One hundred and four implants were placed in 15 patients using a one-stage protocol. After planning the location of all implants via 3-dimensional software (NobleClinician), 4-8 implants were placed in each edentulous arch based on anatomical measurements. Twenty edentulous arches were treated with full arch implant-supported fixed dental prostheses utilizing CAD/CAM milled titanium frameworks . All patients were followed up for 48±4 months. Clinical performance of the implants and restorations were evaluated for implant/prosthesis survival, framework fit, marginal bone levels, and maintenance requirements. One implant was lost during the follow up period, giving an implant survival rate of 99.1%. The average distance from the implant platform to first bone-implant contact was 1.1±0.2 mm from the time of metal-framework try-in to the time of the last recall appointment. None of the prostheses needed a replacement, indicating the prosthesis success rate was 100%. Sixteen occlusal adjustments and 5 broken denture teeth were repaired chairside during the study period. The results of this retrospective clinical report suggest that CAD/CAM milled titanium frameworks using the software and scanner presented in this study fit accurately and can be a viable treatment option to restore edentulous arches.

** Key words:**CAD/CAM, framework, implant, mandible, titanium.

## Introduction

Oral implants have been one of the proposed treatment options for patients missing teeth during the past three decades (1,2). Due to the successful outcome of implant dentistry, many manufacturers and researchers have proposed more innovative and convenient protocols to allow a greater number of clinicians to provide implant treatment for a wider range of patients while maintaining a predictable treatment outcome (1,2). For example, one of the proposed strategies to treat complete edentulism has been implant supported overdentures. One of the recent major developments in implant prosthodontics has been the adoption of engineering principles in the form of computer-aided design and computer-aided manufacturing (CAD/CAM) (2-4).

CAD/CAM production involves three consecutive steps: scanning, CAD modeling, and CAM production (2-4). The scanner is the data acquisition system that records the 3-dimensional (3-D) geometry of the infrastructure and converts the actual dental model into a virtual dental model. The CAD component virtually designs the 3-D contour of the final component. The CAM system produces the actual component according to the virtual design (2-4). In implant dentistry, the frameworks are produced by milling at a central production facility (2-4).

The many benefits associated with CAD/CAM dentistry include: precision of fit, durability, simplicity, cost effectiveness, manufacturing and esthetic material application (1-3). Thus, computer-aided design and computer-aided manufacturing has become increasingly popular for restoring patients with partial or complete edentulism (1-3).

The aim of this report is to analyze the four-year clinical performance of 20 CAD/CAM frameworks supporting full arch implant-supported screw-retained fixed dental prostheses in 15 patients.

## Case Report

Fifteen patients with at least one edentulous arch who desired fixed restorations presented to the University of Texas, School of Dentistry clinics, where the first author used to practice. The major concerns of these patients were the poor retention and esthetics with their existing removable dentures. Institutional Review Board approval has been obtained for this analysis.

Patients were included in the report after careful review of their clinical and radiographic findings. 3-D implant planning software was used to place 4-8 implants in each edentulous arch (NobelClinician, NobelBiocare USA, Yorba Linda, CA). Each patient signed an informed consent before implant placement. Then by utilizing a one-stage surgical approach, all 104 implants (NobelReplace Straight/Tapered Groovy, Nobel Biocare, Yorba Linda, CA) were placed and monitored for healing for 3 months in the mandibles and 6 months in the maxillae.

**Figure 1:**
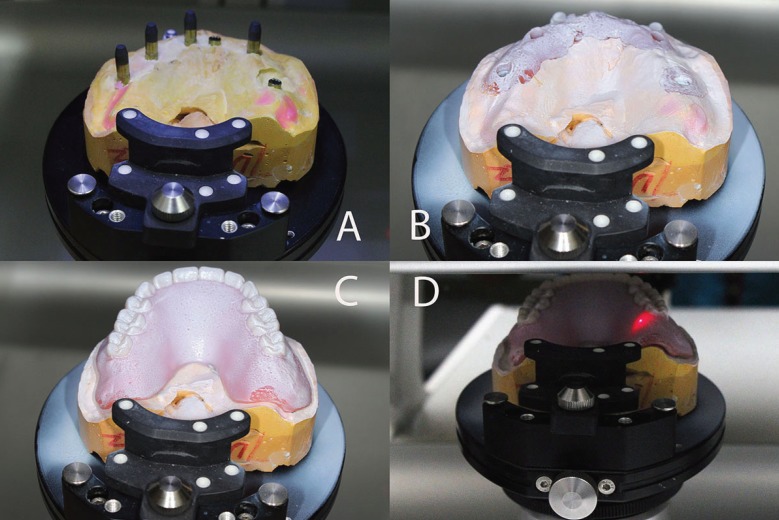
The scanning abutments were screwed on the implant replicas and scanned to locate the implant platforms (A). After the application of zinc-oxide powder spray, the definitive cast (B) to register the tissue surface and the trial denture on the definitive cast were scanned (C,D).

**Figure 2:**
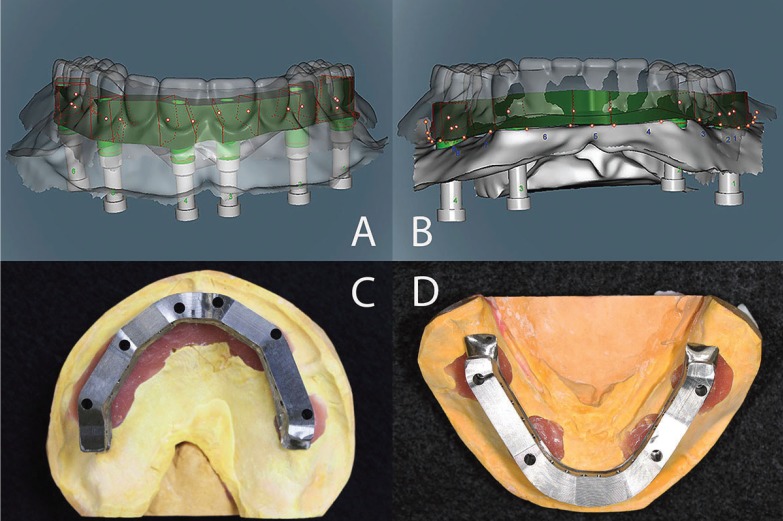
Based on the scan of the trial denture, contour and dimension of the frameworks were determined (A,B). Occlucal views of the milled frameworks (C,D).

The steps below were followed to fabricate each implant-supported, screw-retained FPD. After unscrewing the healing abutment, the impression copings were screwed onto the implants. By using impression copings (Nobel Biocare USA, Yorba Linda, CA), and vinyl polysiloxane impression material (Aquasil, Dentsply Intl, York, PA) an implant-level impression was made. Implant replicas (Nobel Biocare USA, Yorba Linda, CA) were attached to the impression copings, and the definitive casts were made using Type IV dental stone (ResinRock, Whip Mix Corp, Louisville, KY). Through utilization of traditional prosthetic methods, maxillary and mandibular trial dentures were fabricated and a trial denture insertion was performed to evaluate centric occlusion, esthetics, phonetics, and occlusal vertical dimension. Zinc-oxide powder was used to spray each definitive cast, and then the scanning abutments were screwed on the implant replicas (Fig. 1). This allowed the software to locate the implant platforms. The definitive cast without scanning abutments was also scanned to enable the software to register the tissue surface. Then, zinc-oxide powder spray was used to scan each trial denture on the definitive cast. If the scanning process was performed accurately, the planning software superimposes these three different scans without any problems. Based on the scan of the trial denture, contour and dimension of the frameworks were determined (Figs. 2A,B). Information obtained regarding each arch was sent electronically to the Production Center.

**Figure 3:**
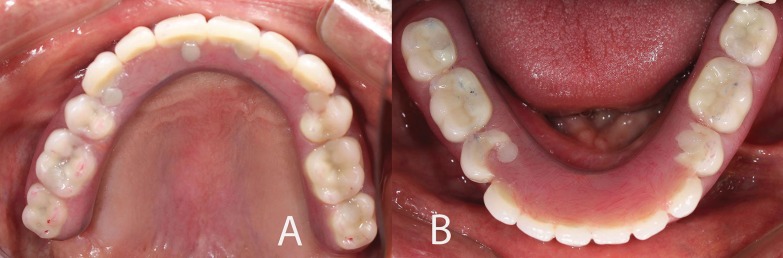
Occlucal views of both restorations at insertion.

Titanium blocks were used to mill complete-arch titanium frameworks by using CAD/CAM technology in the Production Center. Three days post scanning, the frameworks arrived at the clinic (Figs. 2C,D). The intra-oral fit (single-screw test and radiographs) of the frameworks was verified after confirming the fit of each titanium framework on the definitive cast. The denture teeth were transferred from the first trial denture to the framework and a second trial denture insertion was performed to confirm the centric occlusion, esthetics, phonetics, and occlusal vertical dimension. The implant-supported, screw-retained FPDs were processed, finished, and polished in the laboratory and then screwed on the implants in the clinic. Cotton pellets and composite resin were used to cover the screw access holes (Fig. 3).

## Results

Fifteen patients (9 females, 6 males), whose ages ranged from 52 to 79 years (mean age 70±8 years) received 104 implants to support 20 full-arch fixed dental prostheses using titanium frameworks milled by a CAD/CAM method. The patients were followed-up for 48±4 months. One implant was lost before the prosthesis insertion, indicating the implant survival rate of 99.1 %. The average distance from implant platform to bone-implant contact was 1.1±0.2 mm from the time of the metal-framework try-in to the time of last recall appointment. Periapical radiographs using the paralleling technique were taken at the framework try-in appointment and at the last recall appointment. The average of mesial and distal marginal bone changes for all implants were recorded by using the distance measurement tool incorporated in the software (Axium, British Columbia, Canada).

All frameworks fit accurately, which were verified by the single screw test and digital radiographs. No sectioning or welding was needed for any framework. During the follow up period, 16 occlusal adjustments were made without removing the prostheses, and 5 broken/detached denture teeth were attached back on the framework using auto-polymerizing acrylic resin. Twelve sore spots were relieved in opposing removable complete dentures. Two abutment screws were replaced as they loosened twice. None of the prostheses/frameworks needed a replacement, indicating a prosthesis success rate of 100%.

## Discussion

In this analysis, the fabrication procedure for implant-supported FPDs using CAD/CAM technology and 3-D design software has been described, and the outcomes of these patients presented. The first advantage of this technique is precision fit of the framework which minimizes biological and mechanical complications due to strain development and gap formation (5).

Implant CAD/CAM frameworks have been reported to be consistently better fitting than conventional cast components due to minimal human intervention and not requiring several fabrication steps such as waxing and investing (6,7). It has been noted in previous studies that fabrication steps ranging from impression, to cast pouring, wax-up, investing, metal casting and polishing lead to further inaccuracy in the final framework (6,7). Instead of waxing and casting, the entire CAD/CAM process is fully automated following the scanning step which eliminates many error-introducing fabrication steps. The other important advantage is the significant reduction in laboratory/technical time and involvement (5,7). A well-designed implant CAD/CAM metal framework rarely requires additional intervention by the dental technician.

With regards to marginal fit of CAD/CAM frameworks, few studies are available in the literature (8-10). However, direct comparisons between this study and other studies may not be made, due to discrepancies in types of designs, implants, software and scanners used by the previous studies. Ortorp and Jemt, used CNC titanium frameworks and cast gold-alloy frameworks in the edentulous jaw (10). The cumulative survival rates of the prosthesis and implants were 95.6% compared with 98.3%, and 95.0% compared with 97.9% for test and control groups after 10 years, respectively. One prosthesis was lost in each group due to loss of implants, and one prosthesis failed due to framework fracture in the test group (10). In the present study, the implant survival rate was 99.1% and the average distance from implant platform to bone-implant contact was 1.1±0.2 mm. There was no need for any prosthesis replacement which gives a procedure success rate of 100%.

It should be noted that this procedure is technique sensitive and requires the operator to have certain expertise, training and knowledge to utilize the software and hardware accurately to prevent possible costly failures (11). Even though the involved steps were explained in this report, the readers need to ensure they have sufficient knowledge and proper armemantarium before making any attempt to perform this type of procedure.

## Conclusions

The outcomes of this retrospective report have suggested that the CAD/CAM milled titanium frameworks can be used as an alternative to cast frameworks to restore edentulous arches. However, it is recommended that only clinicians and dental technicians with adequate knowledge and experience should involve in this treatment due to steep learning curve.
